# Lipoprotein Ratios as Surrogate Markers for Insulin Resistance in South Indians with Normoglycemic Nondiabetic Acute Coronary Syndrome

**DOI:** 10.1155/2014/981524

**Published:** 2014-05-18

**Authors:** Medha Rajappa, M. G. Sridhar, J. Balachander, K. R. Sethuraman, Kalai Selvi Rajendiran

**Affiliations:** ^1^Department of Biochemistry, Jawaharlal Institute of Postgraduate Medical Education and Research, Puducherry 605 006, India; ^2^Department of Cardiology, Jawaharlal Institute of Postgraduate Medical Education and Research, Puducherry 605 006, India; ^3^Department of Medicine, Jawaharlal Institute of Postgraduate Medical Education and Research, Puducherry 605 006, India

## Abstract

*Background*. Insulin resistance has been associated with dyslipidemia and cardiovascular disease. Even though homeostasis model assessment of insulin resistance (HOMA-IR) is a well-known insulin resistance predictor, estimation of serum lipoprotein ratios has been recently suggested as a surrogate marker for insulin resistance. Here, we evaluated the relationship between lipoprotein ratios and insulin resistance in normoglycemic nondiabetic south Indians with acute coronary syndrome. *Methods*. 100 normoglycemic nondiabetic ACS patients and 140 controls were enrolled in the study. Levels of fasting glucose, fasting insulin, and lipid profile [total cholesterol (TC), triglycerides (TG), and high density lipoprotein cholesterol (HDL-C)], lipoprotein(a) [Lp(a)] levels were measured and lipoprotein ratios were computed. HOMA-IR was used to calculate the insulin resistance. Receiver operating characteristic curves (ROC) analysis was used to compare the power of these lipoprotein ratios to predict insulin resistance. *Results*. Lipoprotein ratios were significantly higher in normoglycemic nondiabetic ACS patients, as compared to healthy controls, and were significantly correlated with HOMA-IR by Spearman's rank correlation analysis. ROC curve showed that Lp(a)/HDL-C and TG/HDL-C ratios were the best surrogate predictors of insulin resistance in normoglycemic nondiabetic ACS. *Conclusion*. This study demonstrates that serum lipoprotein ratios significantly correlate with insulin resistance in normoglycemic nondiabetic ACS. Lp(a)/HDL-C and TG/HDL-C ratios could be used as surrogate markers of insulin resistance in atherosclerosis-prone south Indians with normoglycemic nondiabetic ACS.

## 1. Introduction

Coronary artery disease (CAD) has been reported as the first leading cause of death in South Asia, accounting for 13.6% of all deaths [1]. Also, there are extreme severity and prematurity of acute coronary syndromes (ACS) in Asian Indians who were found to have high mortality from this disease specifically from the Indian subcontinent [2]. Insulin resistance (IR) has been suggested as a risk factor for developing ACS [3]. Lee et al. have reported that despite insulin resistance being associated with conventional risk factors, it plays a role in the evolution of coronary atherosclerotic plaques in asymptomatic subjects [4]. Insulin resistance seems to be a significant risk factor for coronary events [5]. Plasma triglyceride (TG) level, independently associated with insulin resistance and hyperinsulinemia, is an independent predictor of CAD [6]. A previous study has shown that TG/HDL-C ratio is a significant predictor of cardiovascular disease [7]. Lipoprotein(a) [Lp(a)] also correlates with coronary artery disease with insulin resistance such as type 2 diabetes mellitus [8].

The use of simple ratios to identify insulin resistance would be of immense clinical use, as surrogate markers for insulin resistance. The current methods to evaluate insulin resistance involving sophisticated methodology are expensive and time-consuming. Hence, there is an urgent need to develop a relatively simple and economic method to investigate insulin resistance in ACS. We aimed to explore the utility of simple lipoprotein ratios as surrogate markers in the diagnosis of insulin resistance in patients with normoglycemic nondiabetic patients with ACS.

## 2. Methods

This hospital-based case-control study included 100 normoglycemic nondiabetic ACS patients presenting to the medical intensive care unit (MICU) of the hospital, within 12 hours of onset of symptoms, with or without ECG changes, and 140 healthy controls. The study protocol was approved by the Institute of Ethics Committee (Human Studies). Acute coronary syndrome was diagnosed based on clinical, electrocardiographic, and biochemical criteria. The patients were enrolled in the study group after giving informed consent and filling in a structured questionnaire, including details of classical risk factors such as family history of CAD, hypertension, and smoking. Also enrolled, after giving informed consent, were 140 nondiabetic, healthy controls who satisfied the following criteria: normal glucose tolerance test, absence of angina (Rose questionnaire), absence of history of any vascular disease [acute myocardial infarction (AMI), stroke, or intermittent claudication], normal 12-lead resting electrocardiograms, and no past history of any cardiac illness, hypertension, diabetes mellitus, and other metabolic illnesses.

The present study did not include patients or control subjects with a history of endocrine, metabolic, neoplastic, hepatic, renal, infectious, autoimmune, or peripheral arterial disease, pregnancy, and surgical correction in preceding 6 months or those on any medication such as anti-inflammatory or lipid lowering drugs. After written informed consent was obtained from all study subjects, a fasting blood sample was taken within 12 hours of presentation to the hospital and plasma was separated.

Plasma glucose, total cholesterol, triglyceride and HDL-cholesterol (after LDL precipitation with heparin-MnCl_2_) were estimated on the same day using commercial kits on the 550 Express autoanalyzer. The intra-assay and interassay CVs were 4% to 6%, within the recommended range suggested by the National Cholesterol Education Program [9]. LDL-cholesterol was calculated using Friedewald's formula [10]. The remaining plasma was stored at −80°C, until the assays were performed. Lp (a) was determined quantitatively by “INNOTEST Lp(a) ELISA” (Innogenetics NV, Belgium). Insulin was determined by radioimmunoassay (RIA) (radiopharmaceuticals and labelled compounds, Board of Radiation and Isotope Technology, BARC, Vashi Complex, NAVI, Mumbai, India). Insulin resistance indices were calculated by the formula: HOMA IR = [fasting glucose (mg/dL) × fasting insulin (*μ*IU/mL)]/405 [11]. Patients with HOMA-IR ≥ 2 were defined as insulin resistant. Patients with HOMA-IR < 2 were classified as insulin sensitive.

## 3. Statistical Analysis

Baseline characteristics of cases and controls were analyzed using descriptive statistics. The normality of continuous data was assessed by Kolmogorov-Smirnov test. The data were described as mean ± standard deviation and compared by Mann-Whitney* U* test. Association of risk factors between the two groups was assessed by chi-square test. Correlation between biochemical parameters was studied using Spearman's rank correlation test. To determine which lipoprotein ratio best predicts the insulin resistance (HOMA > 2), a receiver operating characteristic curve (ROC) was constructed and the area under the curve was calculated. Analysis was carried out at 5% level of significance and *P* < 0.05 was considered as statistically significant. Statistical analysis was performed using IBM SPSS statistics version 20 for windows.

## 4. Results

Table 1 showed the baseline characteristics of the study groups. BMI and systolic and diastolic BP were significantly higher in the ACS patients, as compared with controls. 67% of patients had Q-wave AMI, 31% had unstable angina, and 2% had non-Q wave AMI.

Table 2 shows the association of risk factors in nondiabetic ACS patients and controls. The risk factors were significantly higher in patients with nondiabetic ACS, as compared with controls.

Routine biochemical analytes are depicted in Table 3. Fasting glucose, fasting insulin, HOMA-IR, lipid parameters (other than HDL-c), lipoprotein(a) and cardiac enzymes, creatine kinase (CK), CK-2/CK-MB, and AST were significantly higher in nondiabetic ACS than in controls (*P* < 0.01).

Lipid indices such as non-HDL-C, TC/HDL-C, LDL-C/HDL-C, TG/HDL-C, non-HDL-C/HDL-C, and Lp(a)/HDL-C were significantly higher (*P* < 0.001) in nondiabetic ACS patients than in controls (Table 4). Correlation of all these lipoprotein ratios with HOMA-IR is shown in Table 5, where all lipoprotein ratios correlated significantly.

**Figure 1:**
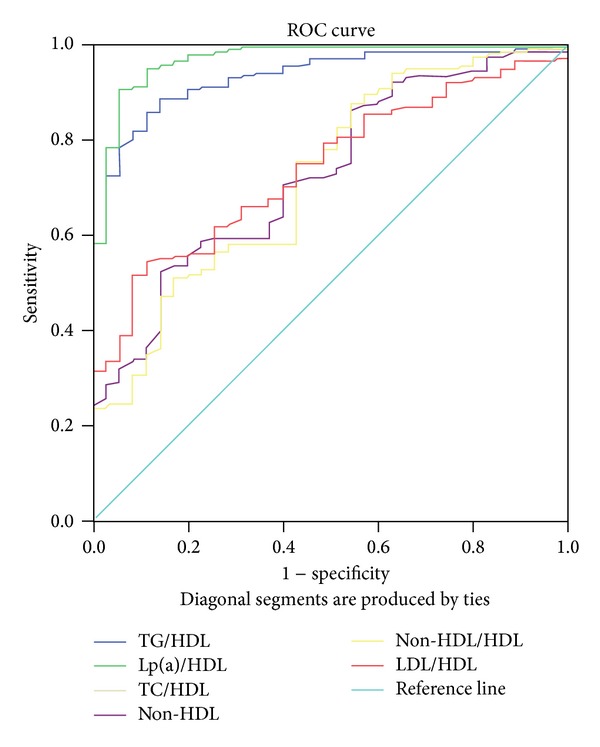
ROC curve for the lipid indices for the insulin resistance prediction.

Figure 1 depicts the ROC curve for the lipid ratios to detect insulin resistance.  Table 6 showed area under curve of the lipid ratios indices for prediction of insulin resistance in normo-glycemic non-diabetic ACS patients. Area under the curve was maximum for the two ratios, TG/HDL-C (0.933) and Lp(a)/HDL-C (0.968), for prediction of insulin resistance in nondiabetic ACS. Among all lipid indices, Lp(a)/HDL-C was the best predictor and showed sensitivity of 96.6% and specificity of 82.9% with cut-off value of 0.295. TG/HDL-C showed sensitivity of 88.8% and specificity of 82.9%, with cut-off value of 1.463, as shown in Table 7.

## 5. Discussion

Our study population constituted the CAD-prone south Indian population, who showed a significant elevation in the HOMA-IR index in normoglycemic nondiabetic ACS patients when compared to the controls. This is in accordance with a previous study by Lazerri and coworkers in nondiabetic STEMI patients [12]. Association of insulin resistance with lipoprotein ratios has been shown in patients with type 2 diabetes mellitus [13].

Insulin resistance is characterized by not only decreased glucose utilization by tissues in response to insulin but also myriad of events that increase significantly the risk for cardiovascular disease [14]. The multifactorial pathogenesis of insulin resistance syndrome and associated atherogenic dyslipidemia is very complex. Metabolic dysregulation of fatty acids is at the heart of the pathophysiology of the insulin resistance syndrome [14].

Although HOMA-IR is gaining wide acceptance as a measure for insulin resistance, there is no consensus regarding its cut-off value for identification of insulin resistance [15]. This is true for Indian subjects also. In our study, insulin resistance was identified as HOMA-IR ≥ 2 and insulin sensitivity was identified by HOMA-IR < 2. This was in line with earlier studies by Sinha et al. [16] and Ray et al. [17].

Several studies have demonstrated that the TC/HDL-C and the LDL-C/HDL-C ratios are better predictors of atherosclerosis and cardiovascular disease than single lipid markers [18–21]. In our study, all the lipid ratios were elevated in the group of normoglycemic nondiabetic ACS, as compared with controls, and correlated significantly with insulin resistance, as measured by HOMA-IR index.

In normoglycemic nondiabetic ACS patients who had HOMA-IR > 2, all lipoprotein ratios had significant ability of detecting insulin resistance shown by area under curve; however, the best predictors of insulin resistance were Lp(a)/HDL-C ratio, which showed sensitivity of 96.6% and specificity of 82.9% with cut-off value of 0.295, and TG/HDL-C ratio which showed sensitivity of 88.8% and specificity of 82.9%, with cut-off value of 1.463. In contrast to our findings, a study in the African American population showed that TG/HDL-C was not a reliable marker for insulin resistance [21].

Lp(a), an important genetic contributor in the progress of myocardial infarction and other cardiovascular diseases, has been shown to correlate with clinically expressed ACS [22]. In our study, Lp(a)/HDL-C ratio was the best predictor and surrogate marker for insulin resistance in normoglycemic nondiabetic ACS patients.

A recent study by Ray et al. has showed TG/HDL-C and TC/HDL-C as insulin resistance markers in the ACS patients with impaired fasting glucose [17]. Our study included ACS patients who had normal glycemic levels and we also included Lp(a), along with the lipid profile parameters in the lipid ratios for prediction of insulin resistance, as south Indians are genetically known to have higher preponderance for CAD and higher Lp(a) levels and high incidence of central obesity and insulin resistance [23–27].

Insulin resistance syndrome, predominantly found in Indians, is associated with increased atherothrombosis [12]. Insulin resistance syndrome produces a prothrombotic state due to the stimulatory effects of insulin on smooth muscle proliferation and migration of smooth muscle from media to intima and the increased levels of platelet activator inhibitor-1 (PAI-1) [14]. Lp(a) also contributes to the disease progression in CAD as it leads to the upregulation of PAI-1, which is known to be elevated in insulin resistance states [8]. All these effects may be potentiated by concomitant dyslipidemias. This probably explains the highly significant positive correlation of Lp(a)/HDL ratio with insulin resistance in the present study.

A major limitation of the present study was that we used HOMA-IR as an estimate of insulin resistance and not the “gold-standard” method hyperinsulinemic hypoglycemic clamp technique. If we had used this technique, it would have validated the findings further.

We conclude that the Lp(a)/HDL-C and TG/HDL-C ratios could be used as surrogate measures of insulin resistance in normoglycemic nondiabetic CAD-prone south Indians with ACS. Since Lp(a) is a nonmodifiable genetic risk factor, there is a need to create awareness for early detection and modification of other risk factors in young individuals, to prevent the early onset of insulin resistance and premature CAD in south Indian population.

## Figures and Tables

**Table 1:** Baseline characteristics of patients with ACS and controls.

Parameters	Cases (*n* = 100) mean ± SD	Controls (*n* = 140) mean ± SD	*P* value
Age (years)	51.03 ± 12.59	51.16 ± 11.85	0.422
Gender (male : female)	85 : 15	111 : 29	—
BMI (kg/m^2^)	25.83 ± 5.61	21.83 ± 2.30	<0.01
W/H ratio	0.942 ± 0.06	0.938 ± 0.06	0.446
Systolic BP (mm Hg)	130.86 ± 16.72	114.39 ± 9.2	<0.01
Diastolic BP (mm Hg)	85.40 ± 13.65	75.02 ± 7.7	<0.01
Unstable angina (%)	31	—	—
Non-Q wave MI (%)	2	—	—
Q-wave acute MI (%)	67	—	—

**Table 2:** Association of risk factors between patients of nondiabetic ACS (*n* = 100) and controls (*n* = 140) by chi-square test.

Risk factors	Cases *n* (%)	Controls *n* (%)	*χ* ^2^ value	*P* value
Smoking	72 (72)	29 (20.71)	62.95	<0.01
Hypertension	29 (29)	—	46.18	<0.01
LVH	12 (12)	—	19.242	<0.01
Obesity	51 (51)	8 (5.7)	64.52	<0.01
Postmenopausal	15 (15)	19 (13.57)	7.46	0.024
High TG	37 (37)	—	61.241	<0.01
High LDL cholesterol	62 (62)	—	117.0	<0.01
High total cholesterol	51 (51)	—	90.67	<0.01
Low HDL cholesterol	62 (62)	—	117.0	<0.01
Alcoholism	24 (24)	5 (3.57)	22.92	<0.01
Family history of CAD	39 (39)	—	65.14	<0.01
Past history of CAD	19 (19)	—	28.88	<0.01

**Table 3:** Comparison of biochemical parameters between nondiabetic ACS and controls.

Parameters	Cases (*n* = 100) mean ± SD	Controls (*n* = 140) mean ± SD	*P* value (Mann-Whitney *U* test)
Fasting glucose (mg/dL)	76.8 ± 5.5	66.1 ± 7.0	0.034
Insulin (µIU/mL)	19.9 ± 3.7	13.5 ± 2.7	0.012
HOMA-IR	3.8 ± 1.3	2.6 ± 0.7	0.033
Total cholesterol (mg/dL)	225.2 ± 40.1	155.4 ± 26.0	<0.001
LDL cholesterol (mg/dL)	149.0 ± 33.1	88.0 ± 23.50	<0.001
VLDL cholesterol (mg/dL)	32.7 ± 10.7	16.4 ± 6.0	<0.001
HDL cholesterol (mg/dL)	43.9 ± 16.09	50.87 ± 10.29	<0.001
TG (mg/dL)	162.3 ± 46.1	82.23 ± 28.5	<0.001
Lipoprotein(a) (mg/dL)	68.2 ± 22.2	17.9 ± 6.20	<0.001
Total CK (IU/L)	631.3 ± 648.9	56.6 ± 28.9	<0.01
CK-2/CK-MB (IU/L)	77.70 ± 64.5	14.02 ± 6.7	<0.01
AST (IU/L)	290.8 ± 363.0	31.7 ± 34.1	<0.01

**Table 4:** Comparison of lipid indices in study populations.

Parameters	Cases (*n* = 100) mean ± SD	Controls (*n* = 140) mean ± SD	*P* value (Mann-Whitney *U* test)
Non-HDL-C (mg/dL)	189.1 ± 45.0	104.5 ± 28.5	<0.001
TC/HDL-C	7.0 ± 3.9	3.2 ± 0.9	<0.001
LDL/HDL-C	4.7 ± 3.0	1.8 ± 0.7	<0.001
TG/HDL-C	5.1 ± 3.5	1.7 ± 0.7	<0.001
Non-HDL-C/HDL-C	6.0 ± 3.9	2.2 ± 0.9	<0.001
Lp(a)/HDL-C	2.1 ± 1.1	0.4 ± 0.1	<0.001

**Table 5:** Correlation coefficients of lipid indices with HOMA-IR by Spearman's rank correlation analysis.

Lipid indices	*r* value	*P* value
Non-HDL-C	0.635	<0.001
TC/HDL-C	0.640	<0.001
LDL/HDL-C	0.641	<0.001
TG/HDL-C	0.789	<0.001
Non-HDL/HDL-C	0.640	<0.001
Lp(a)/HDL-C	0.868	<0.001

**Table 6:** Area under the ROC curve for the insulin resistance in nondiabetic ACS.

Lipid indices	AUC ± SE	95% CI	*P* value
Non-HDL-C	0.730 ± 0.043	(0.646–0.814)	<0.001
TC/HDL-C	0.723 ± 0.046	(0.633–0.813)	<0.001
LDL/HDL-C	0.741 ± 0.038	(0.667–0.815)	<0.001
TG/HDL-C	0.933 ± 0.018	(0.897–0.969)	<0.001
Non-HDL-C/HDL-C	0.723 ± 0.046	(0.633–0.813)	<0.001
Lp(a)/HDL-C	0.968 ± 0.014	(0.940–0.996)	<0.001

SE: standard error; CI: confidence interval.

**Table 7:** Cut-off points corresponding to the highest percentage of sensitivity and specificity calculated from ROC curves for the detection of insulin resistance in nondiabetic ACS.

Lipid indices	Cut-off points	Sensitivity (%)	Specificity (%)
Lp(a)/HDL-C	0.295	96.6	82.9
TG/HDL-C	1.463	88.8	82.9
